# Chemoinformatic Analysis of GRAS (Generally Recognized as Safe) Flavor Chemicals and Natural Products

**DOI:** 10.1371/journal.pone.0050798

**Published:** 2012-11-30

**Authors:** José L. Medina-Franco, Karina Martínez-Mayorga, Terry L. Peppard, Alberto Del Rio

**Affiliations:** 1 Torrey Pines Institute for Molecular Studies, Port St. Lucie, Florida, United States of America; 2 Robertet Flavors, Inc., Piscataway, New Jersey, United States of America; 3 Department of Experimental, Diagnostic and Specialty Medicine, University of Bologna, Bologna, Italy; University of Edinburgh, United Kingdom

## Abstract

Food materials designated as “Generally Recognized as Safe” (GRAS) are attracting the attention of researchers in their attempts to systematically identify compounds with putative health-related benefits. In particular, there is currently a great deal of interest in exploring possible secondary benefits of flavor ingredients, such as those relating to health and wellness. One step in this direction is the comprehensive characterization of the chemical structures contained in databases of flavoring substances. Herein, we report a comprehensive analysis of the recently updated FEMA GRAS list of flavoring substances (discrete chemical entities only). Databases of natural products, approved drugs and a large set of commercial molecules were used as references. Remarkably, natural products continue to be an important source of bioactive compounds for drug discovery and nutraceutical purposes. The comparison of five collections of compounds of interest was performed using molecular properties, rings, atom counts and structural fingerprints. It was found that the molecular size of the GRAS flavoring substances is, in general, smaller cf. members of the other databases analyzed. The lipophilicity profile of the GRAS database, a key property to predict human bioavailability, is similar to approved drugs. Several GRAS chemicals overlap to a broad region of the property space occupied by drugs. The GRAS list analyzed in this work has high structural diversity, comparable to approved drugs, natural products and libraries of screening compounds. This study represents one step towards the use of the distinctive features of the flavoring chemicals contained in the GRAS list and natural products to systematically search for compounds with potential health-related benefits.

## Introduction

Natural products [1], [2] and some food materials designated as “Generally Recognized as Safe” (GRAS) [3], [4], [5] are attractive sources from which to identify molecules with potential health-promoting effects and complement the chemical space of drugs [6], [7]. Flavoring substances in the GRAS list (those that comprise discrete chemical entities) are attracting the attention of researchers to systematically analyze structural and physicochemical properties of these compounds and to explore their potential biological activities [8]. Identification of bioactive molecules in the GRAS collection would suggest potential health benefits when included in the human diet, as well as creating the possibility to explore structural analogues of such compounds. Informative comparisons of the chemical properties of GRAS and pharmaceutical compounds have been reported [8], [9], [10]. However, despite the fact that several GRAS compounds are from natural origin, a direct comparison between GRAS chemicals and natural products databases has not yet been reported.

Natural products are particularly attractive sources from which to identify lead compounds for novel targets [11], [12] such as DNA methyltransferase inhibitors, prohormone convertases [13], [14] and epigenetic targets that are relevant for preventive and therapeutic interventions [15]. We have previously collected natural products databases with chemical structures in the public domain [16]. A comprehensive scaffold analysis revealed that the largest natural products collection analyzed in that work was not the most diverse [16]. It was also found that, in general, natural products databases in the public domain have low molecule overlap. In addition to benzene and acyclic compounds, flavones, coumarins, and flavanones were identified as the most frequent molecular scaffolds [16]. A next logical step, that is reported here, is the analysis of physicochemical properties and comparison of chemical structures using structural fingerprints.

Chemoinformatic analysis of compound libraries provides key information with which to characterize the scaffold content, molecular diversity, and coverage of chemical space [17]. Herein, we report a comparative chemoinformatic analysis of GRAS flavoring molecules and selected natural products databases in the public domain. The chemical databases were compared using diverse criteria including physicochemical properties, substructure, atom counts, and structural fingerprints. Results are discussed in the light of previous analyses reported for natural product databases [12], [18], [19], [20], [21], [22], [23].

## Methods

Table 1 summarizes the compound libraries analyzed in this work. Two distinct natural product databases with different numbers of chemical structures were downloaded (August, 2012). We considered a set of 2244 compounds based on the FEMA GRAS list, complete thru GRAS 25 [24]. This was compiled in-house by manually writing out chemical structures (in the case of discrete chemical entities) and transcribing them into SMILES format. An early version of this GRAS database is briefly described in Peppard et al. [25]. Of note, chemical entities in this set could have defined stereochemistry, ambiguous stereochemistry or even be present as racemic mixtures. Therefore, the set of GRAS molecules analyzed in this work contained no stereochemical information and accordingly our chemoinformatic analysis does not include chiral-sensitive descriptors (see below). Two reference databases were used, namely; a collection of 1713 approved drugs obtained from DrugBank [26] and 10000 compounds obtained from Specs World Diversity Set 3 (SpecsWD3) that is a diverse collection of drug-like screening compounds [27]. All structures were standardized and a washing routine, as implemented in Molecular Operating Environment [28], was applied to remove salts and neutralize molecules. Unique structures, as determined by SMILES generated with Molecular Operating Environment, were selected. All calculations of physicochemical properties, ring and atom counts, and structure fingerprints were performed with Canvas software [29], [30].

**Table 1:** Compound databases analyzed in this work.

Database	Source	Size	URL
GRAS	Published FEMA GRAS data	2244	See text
Natural products	Analyticon	2449	http://www.ac-discovery.com
Natural products	Specs	467	http://www.specs.net
Approved drugs	DrugBank	1713	http://www.drugbank.ca
General screening	SpecsWD3	10000	http://www.specs.net

## Results and Discussion

### Physicochemical Properties

**Figure 1:**
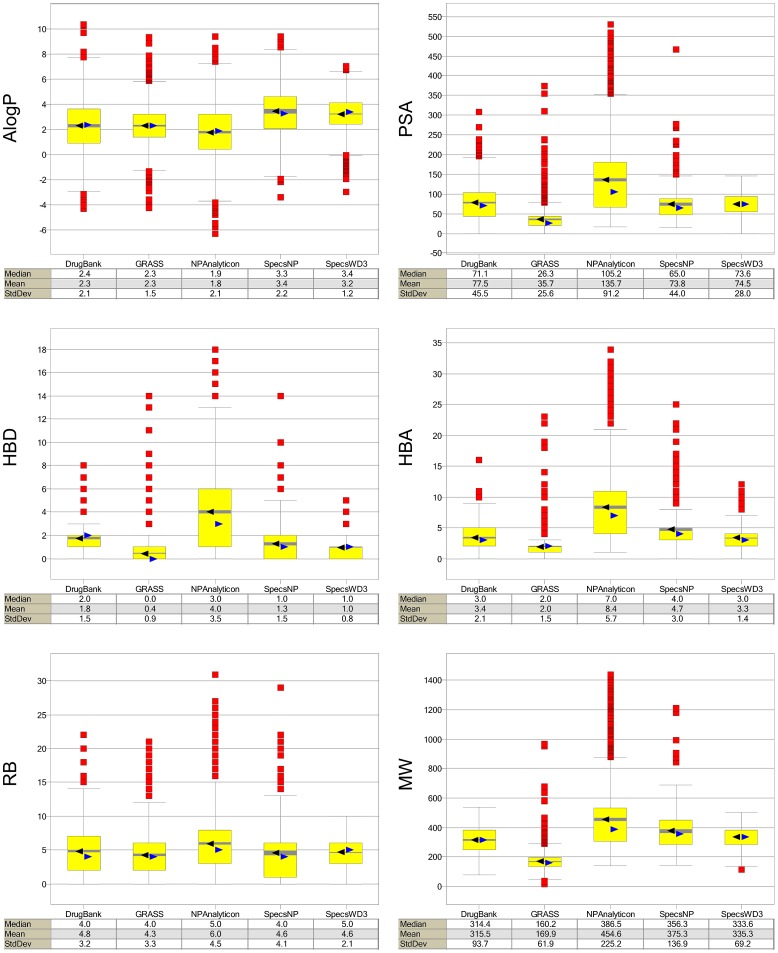
Distribution of six drug-like properties of the databases compared in this work. The yellow boxes enclose data points with values within the first and third quartile; the black and blue triangles denote the mean and median distributions, respectively; the lines above and below indicate the upper and lower adjacent values. The gray band within boxes represents the 95% confidence interval of the mean value (based on the number of data points and StdDev). The red squares indicate outliers. Selected statistics of each distribution are also shown.

The following drug-like properties commonly used to characterize compound collections [21], [31], [32] were computed: the octanol/water partition coefficient (AlogP), polar surface area (PSA), hydrogen bond donors (HBD), hydrogen bond acceptors (HBA), number of rotatable bonds (RB) and molecular weight (MW). Figure 1 summarizes the distribution of the six properties for each database using box-and-whisker plots. The three relevant properties of polarity, flexibility and size are described by AlogP, HBD, HBA, and PSA; RB; and MW, respectively. Figure 1 indicates that natural products from Specs have, in general, properties more similar to drugs than do natural products from Analyticon, except in terms of AlogP. Not surprisingly, the standard deviation for Specs is smaller than that for Analyticon due to the different sizes of the databases (Table 1). As expected, the reference screening collection of drug-like molecules, SpecsWD3, is the most similar to approved drugs, as exemplified by DrugBank. The GRAS compounds are the smallest, least polar, and least flexible cf. any of the five compound databases, as measured by MW, PSA and RB, respectively. Remarkably, the AlogP profile of GRAS compounds is the most similar to drugs (e.g., same mean and very similar median; Figure 1). It has been recognized that, among the properties related to the Rule-of-Five [33], lipophilicity measured by logP is one of the most important metrics in predicting human bioavailability [34]. Interestingly, the distribution of molecular weights of the natural products from Analyticon and Specs is comparable to the natural products implemented in the ZINC database [21] although it is lower than the molecular weight of natural products from other sources [19] including the Traditional Chinese Medicine database [12]. The overall distributions of the physicochemical properties of the GRAS compounds are similar to those previously reported in earlier work (subsets of 1882 and 1736 molecules) [8], [9].

### Distribution of Ring Counts

**Figure 2:**
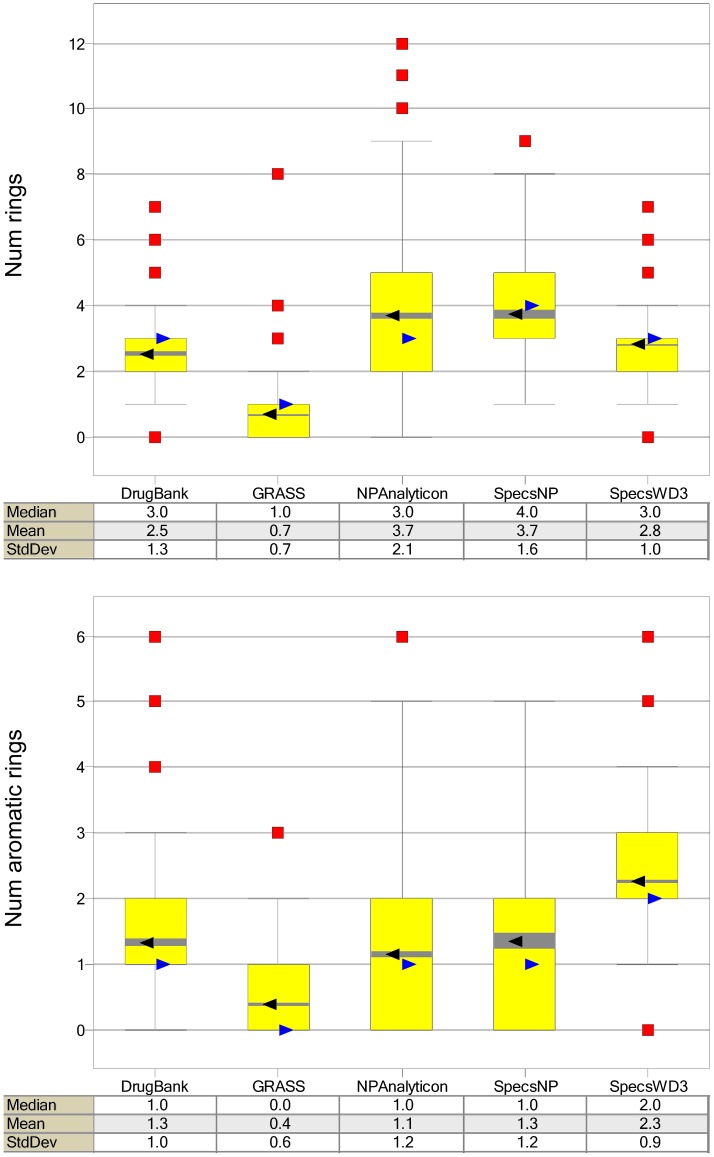
Box-and-whisker plots of the total ring count and aromatic ring count. The symbols and colors in the plots are the same as in Figure 1.

The importance of ring counts in drug development has been discussed extensively [35], [36] and employed to characterize molecular databases [32]. It has been noted that “the fewer the number of aromatic rings contained in an oral drug candidate, the more developable that candidate is likely to be” and that more than three aromatic rings in a molecule correlate with failure in further stages of the development process [35]. More specifically, Ritchie and Mcdonald found more than three rings affect the expected range of values of key properties involved in drug development such as aqueous solubility, lipophilicity, serum albumin binding, cytochrome P450 3A4 isoform inhibition and hERG inhibition. Further details are discussed elsewhere [35]. Figure 2 shows box-and-whisker plots corresponding to the distribution of total numbers of rings and aromatic rings in the present study. The plots in Figure 2 show that the natural products databases have larger numbers of rings cf. the drugs. This is in agreement with previous reports analyzing different databases [19]. In sharp contrast, GRAS compounds have the smallest number of rings (e.g., median of one ring as compared to three or four for drugs, natural products, or the screening collection of drug-like molecules). Similarly, GRAS showed the lowest number of aromatic rings. The mean and median of the aromatic rings for all compound databases analyzed in this work is lower than three suggesting that, in general, all have an acceptable profile to produce developable drug candidate molecules as proposed by Ritchie and Macdonald [35]. Surprisingly, the diverse screening collection SpecsWD3 has, on average, one more aromatic ring than approved drugs and natural products from Analyticon and Specs.

**Figure 3:**
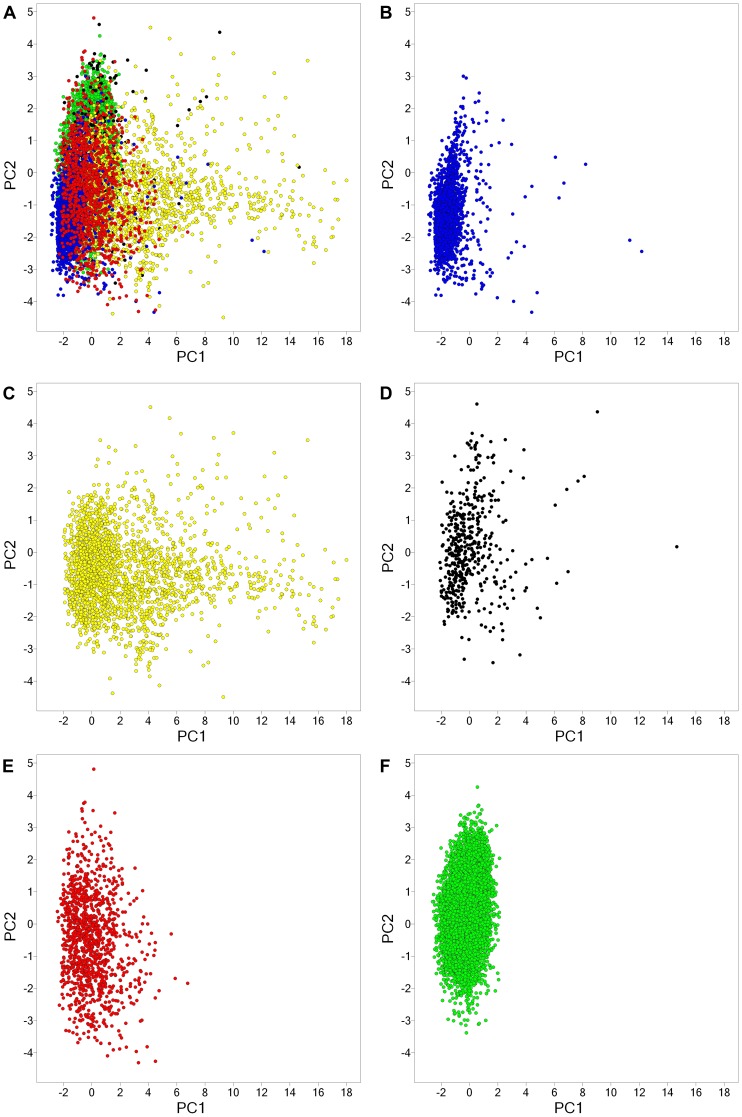
Property space of five databases analyzed in this work obtained by PCA of seven (auto-scaled) molecular descriptors. The first two PCs account for 95% of the variance. The loadings are summarized in Table S1: (A) all libraries; (B) GRAS; (C) natural products - Analyticon; (D) natural products - Specs; (E) DrugBank and (F) SpecsWD3.

**Figure 4:**
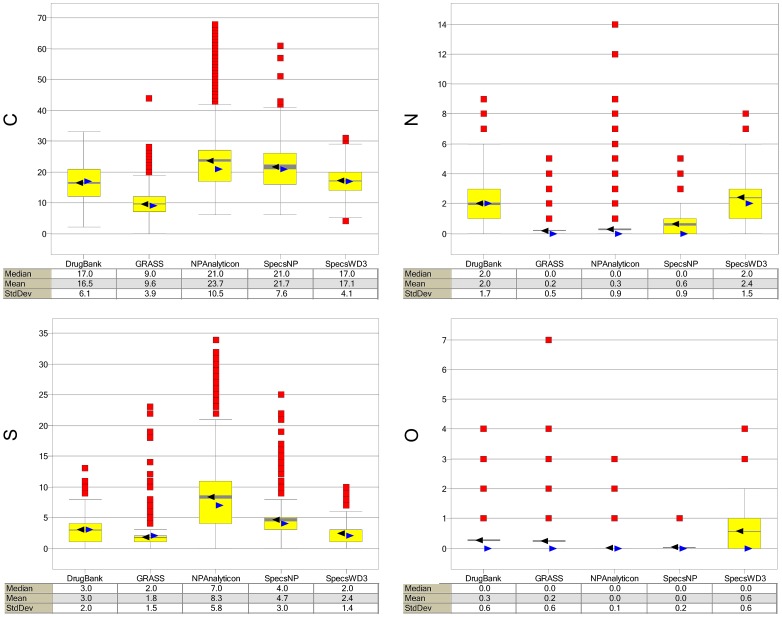
Box-and-whisker plots of atom counts. The symbols and colors in the plots are the same as in Figure 1.

**Table 2:** Summary of the intra-library similarity computed with MACCS keys and radial fingerprints.

Fingerprint	Database	Max	Q3^a^	Median	Q1^b^	Min	U95^c^	Mean	L95^c^	StdDev
MACCS	GRAS	1.00	0.39	0.26	0.16	0.00	0.29	0.29	0.29	0.18
keys	AnalyticonNP	1.00	0.55	0.44	0.34	0.02	0.45	0.45	0.45	0.16
	SpecsNP	1.00	0.47	0.36	0.28	0.06	0.39	0.39	0.39	0.15
	DrugBank	1.00	0.37	0.29	0.22	0.00	0.30	0.30	0.30	0.12
	SpecsWD3	1.00	0.41	0.33	0.26	0.00	0.34	0.34	0.34	0.12
Radial	GRAS	1.000	0.088	0.056	0.032	0.000	0.066	0.066	0.066	0.049
	AnalyticonNP	0.683	0.060	0.045	0.031	0.000	0.048	0.048	0.048	0.026
	SpecsNP	0.662	0.060	0.042	0.025	0.000	0.046	0.046	0.046	0.033
	DrugBank	1.000	0.060	0.044	0.030	0.000	0.047	0.047	0.047	0.026
	SpecsWD3	0.529	0.085	0.067	0.051	0.000	0.070	0.070	0.070	0.027

a Third quartile.

b First quartile.

c 95% confidence of the mean upper (U95) and lower (L95) limits.

**Figure 5:**
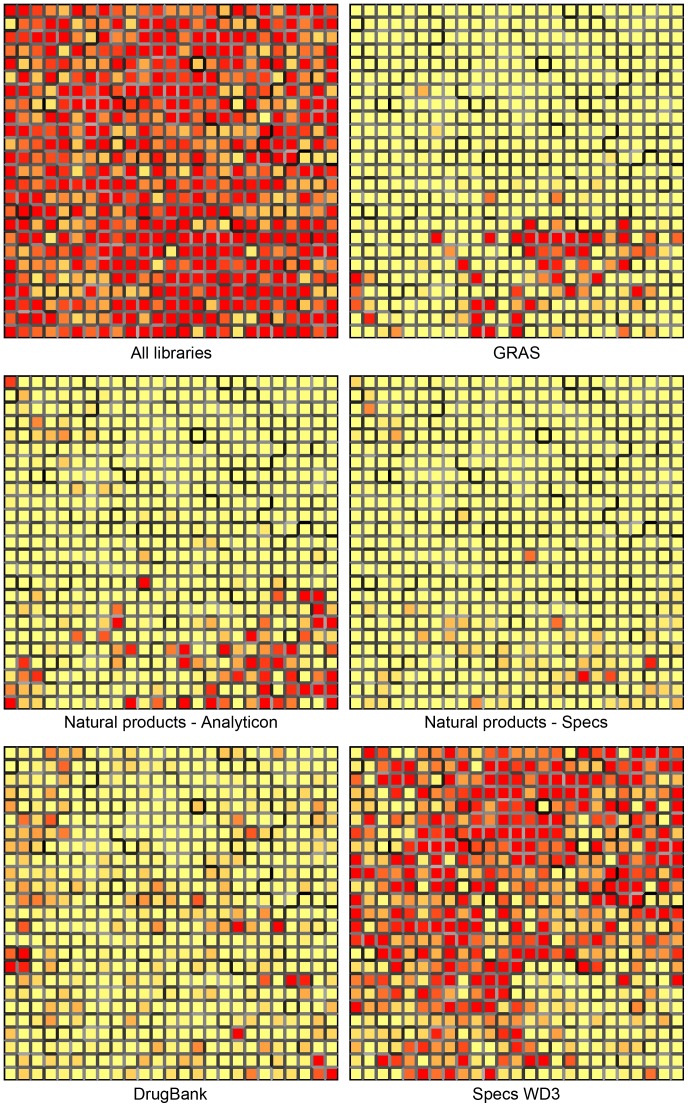
25×25 Self-Organizing-Maps for all five libraries and each library independently. Cells are colored by population, with light yellow for empty cells, and red for cells containing 24 or more compounds. The shading of cell borders indicates the distance between adjacent cells; darker borders indicate larger distance.

**Table 3:** Summary of the comparison between DrugBank and the other four databases computed with MACCS keys and radial fingerprints.^a^

Fingerprint	Database	Max	Q3^b^	Median	Q1^c^	Min	U95^d^	Mean	L95^d^	StdDev
MACCS	GRAS	1.00	0.68	0.58	0.51	0.25	0.61	0.61	0.60	0.14
keys	NPAnalyticon	1.00	0.71	0.63	0.55	0.19	0.65	0.64	0.64	0.14
	SpecsNP	1.00	0.67	0.59	0.52	0.17	0.61	0.61	0.60	0.14
	SpecsWD3	1.00	0.78	0.71	0.67	0.29	0.72	0.72	0.71	0.10
Radial	GRAS	1.00	0.17	0.14	0.11	0.06	0.17	0.16	0.16	0.12
	NPAnalyticon	1.00	0.15	0.12	0.11	0.04	0.14	0.14	0.13	0.07
	SpecsNP	1.00	0.13	0.11	0.09	0.03	0.12	0.12	0.11	0.05
	SpecsWD3	1.00	0.19	0.15	0.12	0.05	0.17	0.16	0.16	0.07

a Statistics correspond to the maximum structure similarity to different compound databases to DrugBank.

b Third quartile.

c First quartile.

d 95% confidence of the mean upper (U95) and lower (L95) limits.

### Visual Representation of the Chemical Space

The number of aromatic rings, along with the six properties (AlogP, PSA, HBD, HBA, RB, MW) discussed above, were employed to generate a visual representation of the chemical space of the five databases. The visualization shown in Figure 3 was obtained by means of principal component analysis (PCA) of the seven auto-scaled properties. The two principal components account for 95% of the variance. Figure 3A shows all libraries within the same coordinates. For visual clarity, Figure 3 also depicts each library individually, again using the same coordinates. Table S1 in the Supporting Information summarizes the corresponding loadings and eigenvalues for the first three PCs. For the first PC, the larger loadings correspond to PSA followed by HBA, whereas for the second PC the largest loadings correspond to AlogP followed by the number of aromatic rings. The high variance explained by the first two PCs (95%) provides a measure of confidence, when analyzing the two-dimensional visualization of chemical space, that it is a reasonable *approximation* of the full seven-dimensions. This visualization shows that the GRAS database covers a broad region of the property space occupied by the drug molecules in DrugBank and the two natural products databases. In addition, several compounds from GRAS densely populate a relatively sparse area of the property space covered by DrugBank and the other compound collections (lower right quadrant of the PCA plot). The different coordinates along PC1 for several GRAS molecules are in agreement with the different profile of primarily PSA, HBA, and number of aromatic rings as compared to other databases (Figure 1 and 2 and Table S1). In Figure 3 it is also remarkable that natural products from Analyticon cover a broad area of the property space that is consistent with the large standard deviation that this collection has in several properties (Figure 1). In contrast, despite the fact that the screening collection SpecsWD3 is the largest database, it covers a much narrower area of the property space (with few if any outliers) which is quite similar to the space of approved drugs. This is in accord with the design of this database that mimics the properties of currently approved drugs.

### Distribution of Atom Counts

Compound databases were further characterized using counts of carbon, nitrogen, oxygen, and sulfur atoms. Similar to the set of physicochemical properties discussed above, this set of atom counts are commonly used to compare compound databases of bioactive molecules and natural products [19]. In addition, the number of carbon, nitrogen, oxygen, and sulfur atoms divided by the number of heavy atoms (i.e., non-hydrogen atoms) in each molecule (i.e., fraction of atom counts) was also analyzed. Figure 4 and Table S2 in the Supporting Information summarizes the results, indicating that natural products from Analyticon and Specs have larger numbers of carbon and, more pronounced, larger numbers and fractions of oxygen atoms as compared to the drugs database. Notably, natural products from Analyticon have a large fraction of oxygen atoms (median and mean of 0.25 and 0.24, respectively) as compared to other collections (median/mean ≤0.17; Table S2). This is in agreement with analysis of other collections of natural products recently reviewed by Lachance et al [37]. Natural products from Specs have a slightly larger number and fraction of nitrogen atoms than do natural products from Analyticon. Both natural products collections have lower numbers and fractions of nitrogen and sulfur atoms cf. the drug database. In fact, natural products have very few sulfur atoms. This is also consistent with the analysis of other natural products databases [37]. In contrast, the reference screening collection SpecsWD3 has similar numbers and fractions of carbon and nitrogen atoms as compared to the drugs database, thought it has, on average, slightly fewer oxygen and slightly more sulfur atoms. The slightly higher number of sulfur atoms in the screening collection can be attributed to the synthetic origin of these molecules that frequently incorporates reagents containing sulfur atoms [19]. GRAS has the lowest number of atom counts and this is not surprising because of the smaller molecular size on average. The fraction of atom counts relative to all heavy atoms (Table S2) reveals, however, that GRAS compounds have a larger fraction of carbon atoms than do the drugs in DrugBank and the screening collection. Similarly, GRAS molecules have a slightly higher fraction of oxygen atoms than molecules in DrugBank and SpecsWD3. GRAS, DrugBank, and SpecsWD3 have similar fraction of sulfur atoms.

### Comparison with Structure Fingerprints

#### Intra-library similarity

The intra-library similarity of the five compound collections was measured using Molecular ACCess System (MACCS) keys (155-bits) and radial fingerprints available in Canvas. The latter are also known as extended connectivity fingerprints [38]. Two representations of different design were employed in order to reduce the well-known dependence of chemical space on molecular representation [39], [40]: MACCS keys are a pre-defined set of 155 structural keys whereas radial fingerprints entail growing a set of fragments radially from each heavy atom over a series of iterations [38]. Table 2 summarizes the statistics of the distribution of all pairwise similarity values computed using the Tanimoto coefficient [41], [42] and the two fingerprint representations. The number of pairwise comparisons range from 108811 in the case of natural products from Specs to 1466328 for DrugBank. For the GRAS database and natural products from Analyticon, and SpecsWD3, random samples of 1000 molecules each (499500 pairwise comparisons) were considered. It has been observed in several studies that random samples of 1000 compounds is generally representative of the structural diversity computed with structure fingerprints [21], [43]. The similarity values computed with MACCS keys and radial fingerprints have different magnitudes, being larger when computed with the former method. This observation is in agreement with a number of previous studies comparing various databases from different sources [40], [44]. Table 2 shows that all five compound databases have high structural diversity, e.g., median and mean MACCS keys/Tanimoto similarity ≤0.45. Among the natural products, the collection from Specs is more diverse than the set from Analyticon (based on MACCS keys). This trend is opposite to the scaffold diversity recently reported where natural products from Analyticon showed larger scaffold diversity than Specs [16]. This result indicates that the larger structural diversity of Specs is due, in part, to the side chains around the core scaffolds. Also, this result is in agreement with the observation that high structural diversity using whole structures does not necessarily correlate with high scaffold diversity. Based on MACCS keys, the GRAS collection showed the greatest structural diversity (median and mean MACCS keys/Tanimoto similarity of 0.26 and 0.29, respectively).

A visual representation of the structural diversity of each database is presented in Figure 5. The figure shows a rectangular MACCS keys-based Kohonen map or self-organizing map [45] generated with Canvas software. The map was applied for all compounds comprising the five libraries in Table 1 (16873 compounds). The self-organizing maps clearly depict the large structural diversity of all databases.

#### Comparison of chemical structures with approved drugs

The molecular databases were also compared to drugs using the same MACCS keys and radial fingerprints employed to measure intra-library similarity. For each compound in DrugBank the maximum MACCS keys and radial similarity were determined and compared to all molecules in the test database using the Tanimoto coefficient. Table 3 summarizes the distribution of 1713 similarity values that represent the similarity of the nearest-neighbor (maximum similarity) of the molecules in a given database to each molecule in the set of approved drugs. Values in Table 3 correspond to the statistics of the nearest-neighbors curves that are frequently used to compare compound databases [12]. Despite the fact that the values have different magnitude (see above), both fingerprints indicate that SpecsWD3 is *relatively speaking* the most similar collection to drugs, as it has the highest similarity values computed using MACCS and radial fingerprints (Table 3). However, although there is at least one molecule in the SpecsWD3 database with MACCS keys and radial fingerprint representation identical to that of a drug compound (as indicated by the maximum similarity of one), most of the compounds from drugs have different chemical structures as indicated by the median, mean and third quartile of the MACCS keys similarity values (Q3 = 0.78). In other words, the nearest-neighbor (closest molecule) of SpecsWD3 to 75% of the drugs has MACCS keys/Tanimoto similarity of 0.78. Based on experience and several reports in the literature, it is well-known that a pair of compounds with MACCS keys/Tanimoto similarity lower than 0.8 can be considered as being dissimilar by a medicinal or experimental chemist. Taken together, the results in Table 3 indicate that all four collections compared to drugs have several molecules with novel chemical structures relative to drugs.

The second closest database to drugs depends on the fingerprint representation; based on MACCS keys, natural products from Analyticon are the second closest compounds to drugs while, based on radial fingerprints, GRAS is the second closest database. Natural products from Specs are, in general, the compounds with the chemical structures most different from drugs. It should be emphasized that the comparison of the databases using structural fingerprints is different from the comparison of the databases using physicochemical properties or ring counts discussed above. Thus, molecules with different chemical structures can have similar properties. In other words, compounds collections can be drug-like in terms of properties but can have novel chemistries. This is clearly illustrated by SpecsWD3 which has, in general, different chemical structures from drugs but similar physicochemical properties.

It is worth mentioning that a deeper understanding of the GRAS and natural compounds collections in terms of their bioactivity profiles might conveniently be obtained using a variety of other computer-aided drug design approaches, in particular docking or pharmacophore screening [46], [47], [48]. In this context, the growing availability of three-dimensional structures of target proteins raises the possibility of deploying these techniques in search of GRAS or natural compounds using ‘target-fishing’ approaches [49]. This is also of clear interest for elucidating the polypharmacological properties of these collections for their role as dietary and nutraceutical components that in many clinical, physiopathological and epidemiological studies have proven to be either detrimental or beneficial to human health.

### Conclusions

We report a comparison of the discrete chemical entities within the FEMA GRAS list of flavoring substances with natural products, approved drugs and a large library of screening compounds using different and complementary criteria. We conclude that GRAS molecules have distinct molecular properties and a lipophilicity profile remarkably similar to approved drugs. The set of GRAS compounds analyzed in this work have a high structural diversity which is comparable to the high structural diversity of drugs, natural products, and screening molecules. Natural products databases from different sources differ in physicochemical properties and structural diversity and this result complements previous conclusions derived from scaffold analysis. These results further emphasize the convenience of using more than one natural product database. By doing so, a broader area in chemical space is covered during experimental and computational screening. Results of this work clearly showed that a compound library with high structural diversity does not necessarily have broad coverage of the property space. The overall low toxicity of the FEMA GRAS list of flavoring substances (considering the quantities and presentations allowed for human consumption), and the well-known relevance of natural products in drug discovery, make these compounds libraries attractive to systematically identify compounds with putative health-related benefits. This work represents a step further in the growing field of Food Informatics [50]. The overall small size of the FEMA GRAS list of flavoring substances raises the question of the relationship between these molecules and other molecular databases such as fragment libraries or collections of lead-like molecules. Such relationships will be fully addressed in a follow up work. Other perspective of this work is to extend the comparison of the chemical databases using additional atom counts, such as halogens, and properties related to absorption, distribution, metabolism, and elimination (ADME).

## Supporting Information

Table S1 Loadings for the first three principal components of the property space of five databases.
(DOC)

Table S2 Counts of fraction of atoms.
(DOC)
