# Supplementary material for: Chemoinformatic Analysis of GRAS (Generally Recognized as Safe) Flavor Chemicals and Natural Products
Source: PLoS One. 2012 Nov 30;7(11):e50798. doi: 10.1371/journal.pone.0050798 (PMC3511266; doi:10.1371/journal.pone.0050798)
Supplement: Table S1 — Loadings for the first three principal components of the property space of five databases. (DOC) [file pone.0050798.s001.doc]

**Table S1.** Loadings for the first three principal components of the property space of five databases (the PCA plot is in Figure 3).

|  | **PC1** | **PC2** | **PC3** |
| --- | --- | --- | --- |
| Eigenvalue | 3.824 | 1.558 | 0.847 |
| Cumulative Variance | 0.814 | 0.949 | 0.989 |
| AlogP | -0.198 | 0.652 | -0.268 |
| HBA | 0.488 | -0.032 | 0.062 |
| HBD | 0.450 | -0.160 | 0.107 |
| MW | 0.436 | 0.313 | -0.017 |
| Num aromatic rings | 0.008 | 0.619 | 0.619 |
| PSA | 0.489 | -0.006 | 0.174 |
| RB | 0.303 | 0.259 | -0.706 |
