# Supplementary material for: Chemoinformatic Analysis of GRAS (Generally Recognized as Safe) Flavor Chemicals and Natural Products
Source: PLoS One. 2012 Nov 30;7(11):e50798. doi: 10.1371/journal.pone.0050798 (PMC3511266; doi:10.1371/journal.pone.0050798)
Supplement: Table S2 — Counts of fraction of atoms. (DOC) [file pone.0050798.s002.doc]

**Table S2.** Counts of fraction of atoms (median/mean/standard deviation).*^a^*

| Fraction*^a^* | GRAS | AnalyticonNP | SpecsNP | DrugBank | SpecsWD3 |
| --- | --- | --- | --- | --- | --- |
| C | **0.82/0.80/0.11** | 0.74/0.75/0.08 | 0.80/0.80/0.07 | 0.75/0.73/0.13 | 0.74/0.74/0.08 |
| N | 0.00/0.02/0.06 | 0.00/0.01/0.03 | 0.00/0.02/0.04 | 0.09/0.10/0.08 | **0.10/0.11**/0.07 |
| **O** | **0.14/0.15/0.10** | **0.25/0.24/0.08** | **0.16/0.17/0.08** | **0.13/0.14/0.10** | **0.10/0.10/0.06** |
| S | 0.00/0.03/0.08 | 0.00/0.00/0.01 | 0.00/0.00/0.01 | 0.00/0.01/0.03 | 0.00/0.03/0.03 |

*^a^* Fraction = Atom count / Number of heavy atoms
